# The Character Position Encoding of Parafoveal Semantic Previews Is Flexible in Chinese Reading

**DOI:** 10.3390/bs15070907

**Published:** 2025-07-04

**Authors:** Min Chang, Yun Ma, Zhenying Pu, Yanqun Zhu, Jingxuan Li, Lvqing Miao, Xingguo Zhu

**Affiliations:** 1 Department of Applied Psychology, School of Education Science, Nantong University, Nantong 226019, China; ntucm@ntu.edu.cn (M.C.);; 2 Department of Traffic Psychology, Institute of Special Environmental Medicine, Nantong University, Nantong 226019, China

**Keywords:** transposed-character effect, semantic preview effect, Chinese reading, eye movement

## Abstract

Extant Chinese studies have documented that transposing characters within two-character words (e.g., 西装 suit) yields greater parafoveal preview benefits for target words compared to replacing the characters with unrelated ones (e.g., 型间 a nonword), i.e., the Chinese character transposition effect. This effect has been interpreted as evidence for flexible positional encoding in parafoveal processing, whereby readers tolerate character order disruptions. Alternatively, it has been attributed to morpheme-to-word activation. The present study aims to further clarify the mechanism of the transposition effect. We manipulated four preview conditions of target words in a sentence, identical, semantic, transposed semantic, and control preview, using an eye tracker to record eye movements. Experiment 1 employed reversible word pairs (e.g., 领带 tie-带领 lead) as semantical and transposed previews for targets (e.g., 西装suit). Experiment 2 used non-reversible word pairs (e.g., 衬衫 shirt-衫衬 a nonword). The results revealed comparable processing for both the semantic and transposed semantic preview conditions. Critically, the transposed semantic preview yielded a processing advantage over the unrelated preview. These findings demonstrated that Chinese readers efficiently extract semantic information from the parafoveal region even when character order is disrupted, indicating flexible character position encoding.

## 1. Introduction

Word recognition, whether in isolation or during sentence reading, begins with orthographic processing. This process involves identifying and encoding the position of constituent letters or characters (Grainger, 2018). Numerous empirical studies have investigated how readers encode the orders/positions of characters/letters in English or Chinese scripts (Chang et al., 2020, 2025; Johnson et al., 2007; Kirkby et al., 2022; Gu et al., 2015, 2023; Gu & Li, 2015; Perea & Carreiras, 2006; Perea et al., 2008, 2012; Rayner et al., 2006; White et al., 2008; H. Yang et al., 2022; for a review, see Grainger, 2024). Consistent findings across studies highlight the transposed-letter/character effect, which has been observed in both the lexical prime paradigm and sentence reading tasks employing the boundary paradigm (Rayner, 1975).

In the lexical masked prime task, a transposed-letter (TL) prime (e.g., “jugde”) or a substituted-letter (SL) condition (e.g., jupbe) is presented before the target word “judge”. Participants show a processing advantage in the TL condition compared to a substituted-letter (SL) condition, i.e., the transposed-letter effect (TLE). This effect has also been observed in sentence reading tasks using the boundary paradigm, where an invisible boundary is placed to the left of the target word in a sentence (Rayner, 1975). This method allows researchers to manipulate parafoveal preview stimuli, such as identical (e.g., 西装), transposed-character (e.g., 装西), or unrelated substituted-character nonwords (e.g., 袭有) of the target word (e.g., 西装). The preview stimulus is replaced with the target word when the reader’s eyes cross this boundary. The processing advantage of transposed-character preview over the substituted-character preview condition is referred to as the transposed-character effect. Such experiments provide valuable insights into the role of orthographic processing and positional encoding during natural reading.

While the transposed-letter effect in alphabetic systems has been extensively researched (for a review, see Grainger, 2024), the landscape of position encoding for Chinese characters remains less well understood. Unlike alphabetic scripts, where letters represent distinct units of meaning, Chinese characters represent morphemes, each carrying its semantic load. Furthermore, the lack of clear spaces between words in Chinese scripts complicates the reading process, as readers must decode continuous character strings without the visual cues typically available in alphabetic languages. Therefore, understanding how Chinese readers process character positions is essential for advancing our knowledge of visual word recognition in logographic writing systems.

Recent psycholinguistic research has robustly demonstrated the transposed-character effect (TC) in both masked priming tasks (Gu et al., 2015) and sentence reading tasks (Chang et al., 2020, 2025; Gu & Li, 2015; J. Yang, 2013; Zhang et al., 2022) in Chinese scripts. Notably, Gu and colleagues established foundational evidence using a masked priming paradigm with two-character Chinese words, manipulating word structure (single-morphemic vs. multiple-morphemic) and priming conditions: identity (IC), transposed-character (TC), and unrelated substituted (SC) conditions. Their results showed a significant TC priming effect, where transposed-character primes (e.g., 啬吝 for target 吝啬) elicited faster lexical decision latencies and higher accuracy than unrelated substituted primes (e.g., 菠汞), while lagging behind identity primes. The effect has since been extended to more complex linguistic units. H. Yang et al. (2019) replicated these findings using four-character Chinese compounds (e.g., 总来的说→总的来说) and demonstrated that the effect persists regardless of text orientation. Specifically, the effect was observed in text presented in both the conventional left-to-right horizontal orientation commonly used in mainland China and the vertical top-to-bottom orientation prevalent in Taiwan and Hong Kong of China. Crucially, the boundary paradigm in sentence reading tasks has yielded particularly compelling evidence for this effect. Chang et al. (2020) reported that transposed-character previews (e.g., “链项” for “项链”) resulted in significantly shorter fixation durations compared to unrelated nonword previews (e.g., “湖姑”), providing direct evidence for the TC effect during natural reading. This finding has been further corroborated in subsequent research with older adults, suggesting the robustness of this phenomenon across different age groups (Chang et al., 2025).

The transposed-character effect in Chinese is commonly attributed to flexible character position encoding during visual word recognition. Alternatively, another explanation framework may also warrant consideration. From the lexical processing perspective, the TC condition preserves the identical morphemic constituents as the target word. According to the Chinese Reader Model (CRM; Li & Pollatsek, 2020), its word processing module simulates parallel activation of all candidate words within the perceptual span, where bottom-up character-level activation feeds forward to whole-word representations. In this framework, the TC condition maintains intact morpheme-to-word associative pathways, whereas the substituted-character conditions do not. Thereby, the observed facilitation in TC conditions could alternatively be explained by preserved character-level activation. Perceptually, TC also exhibits greater visual similarity to the target word than fully substituted characters (Norris et al., 2010), which may further contribute to recognition efficiency.

While two distinct accounts have been proposed to explain the transposed-character (TC) effect in Chinese reading, they may represent complementary rather than competing mechanisms. The conventional explanation emphasizes flexible character order processing during parafoveal preview, while the alternative account highlights the role of morpheme-to-word activation in facilitating word recognition. Crucially, these accounts are not mutually exclusive; rather, the morpheme-to-word activation mechanism may serve as the cognitive foundation that enables flexible character processing in the first place.

The present study aims to advance this theoretical understanding of flexible character order encoding by addressing a critical methodological limitation in prior TC research: the potential confounding of visual similarity between transposed-character previews and their target words. To achieve this, we adopt an experimental paradigm inspired by Peng et al. (1999), who employed reversible word pairs whose transposed forms are also real words (e.g., both 领带 [necktie] and 带领 [lead] are valid words) in a semantic prime paradigm. Their seminal study demonstrated that both the original (领带) and transposed forms of reversible primes facilitated lexical decisions for semantically related targets (e.g., 西装 suit), a phenomenon termed transposed–semantic priming. This finding suggests that reversible word primes simultaneously activate representations of both their original and transposed forms, thereby producing comparable priming effects.

Building upon this theoretical foundation, the current study extends this experimental logic to investigate character position encoding during natural sentence reading. Our current design tries to preclude the influence of activation from the character to the target word. Unlike previous studies, we transposed (e.g., 带领) or substituted (e.g., 阅读) the semantic preview stimuli (e.g., 领带) of the target word but not the target word (e.g., 西装). Using the boundary paradigm (Rayner, 1975), we systematically manipulated four preview conditions for target words: identical preview, semantically related word preview, transposed–semantic related preview word/nonword, and unrelated nonword preview. In Experiment 1, we used reversible word pairs (e.g., 领带 and 带领), as semantically related previews and transposed–semantic related previews to the target word (e.g., 西装), respectively. Experiment 2 utilized non-reversible semantic related words (e.g., 衬衫 T-shirt), whose transposed form (衫衬) constitutes a nonword, to eliminate potential semantic interference from the transposed form. Critically, both transposed–semantic previews and unrelated substituted previews were visually dissimilar to the target word. We hypothesize that transposed–semantic previews will produce similar semantic preview effects to semantically related previews. Furthermore, we predict the transposed–semantic preview condition will yield greater preview benefits compared to the unrelated substituted condition, which would provide compelling evidence for flexible character order encoding during parafoveal processing in Chinese reading.

## 2. General Method

### 2.1. Participants

**Figure 1:**
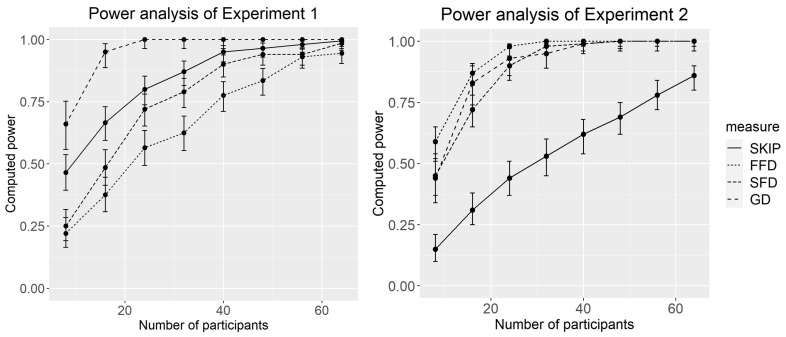
Power estimates for Experiments 1 and 2 for SKIP (skipping rate), FFD (first fixation duration), SFD (single fixation duration), and GD (gaze duration).

To determine the required sample size for our study, we conducted a prior power analysis using the powerSim and powerCurve functions from the simr package (Green & MacLeod, 2016) in R (version 4.0.2, R Development Core Team, 2016). Initially, we performed a pilot study with 24 participants in each experiment, with 4 participants assigned to each of the four lists. Using data from these 24 participants, we fitted mixed-effects models, as described in the Data Analysis section, to obtain the mean, standard deviations, fixed intercept, and slope. The power curves based on these pilot data are shown in Figure 1. The results suggested that a sample size of approximately 50 participants would be sufficient to detect a transposed–semantic preview effect with at least 80% power. However, we anticipated that approximately 20% of the data would be discarded during the preprocessing stage, as detailed in the Data Analysis section. Thus, we decided to recruit 60 Chinese college students (aged 18–25, *M* = 20, female = 42) for the two formal eye-movement experiments. Students who learn Chinese Language and Literature or Psychology were not allowed to participate the experiment. All participants had normal or corrected-to-normal vision and were native Chinese speakers who were unaware of this study’s purpose.

### 2.2. Material and Design

**Figure 2:**
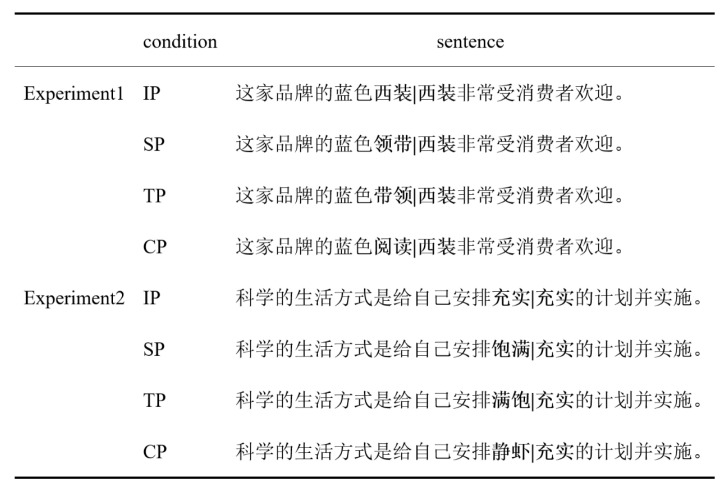
Sample stimuli in Experiments 1 and 2. Note: The “|” represents an invisible boundary, which is preceded by a two-character preview stimulus and followed by a target word. The preview stimuli would transform into the target word 西装 (suit) upon the eye’s saccade from the left to the right of the invisible boundary. The preview stimuli and target words are displayed in bold for illustrative purposes only. During the actual experiments, all words were presented in regular font. The example sentence in Experiment 1 is translated as “The blue suit of this brand is very popular among consumers”. The preview words 西装 (suit), 领带 (tie), 带领 (leading), and 阅读 (reading) were used. In Experiment 2, the example sentence is translated as “A scientific way of life is to make substantial plans and implement these plans”. In place of the words 充实 (substantial) and 饱满 (plump), the nonwords 满饱 and 静虾 were used as preview stimuli. IP, SP, TP, and CP refer to identical preview, semantic preview, transposed–semantic preview, and controlled preview, respectively.

Stimuli consisted of two sets of 80 sentence frameworks, with an example provided in Figure 2. All the sentence stimuli can be found in the Supplement (Tables S3 and S4). The characteristics of each condition are outlined in Table 1. We utilized a within-subjects design with a single factor comprising four levels: identical preview (IP), semantic preview (SP), transposed–semantic preview (TP), and control preview (CP).

### 2.3. Material in Experiment 1

For Experiment 1, following Peng et al. (1999), we chose reversible words as semantic previews according following requirements: (1) The semantic preview (SP) and transposed semantic preview (TP) represent two forms of reversible words, which are semantically unrelated to each other (e.g., 领带_necktie and 带领_lead) and prevent mutual activation at the semantic level, thereby eliminating associations akin to 相互-互相, which means mutual. (2) We excluded words whose pronunciation changes after the transposition of Chinese characters, such as行车-车行, as the character 行 has different pronunciations in 行车 (pronounced as xingche) and 车行 (pronounced as chehang). (3) We excluded monomorphemic words, such as 雅典 (“Athens”).

The semantic relatedness between the target word and each preview condition (SP, TP, and CP) was evaluated on a 5-point scale (from 1 = very unrelated to 5 = very related) by 22 participants. The mean semantic relatedness scores between the target word and the SP, TP, and CP conditions were 3.57 (*SD* = 0.70), 2.1 (*SD* = 0.52), and 1.34 (*SD* = 0.28), respectively, with the following pattern: SL > TL > CL (*t*s < 0.001). Additionally, the semantic relatedness between the SP and TP conditions was assessed on the same 5-point scale by 12 participants. Sentences with semantic relatedness exceeding 2.5 were excluded, resulting in a mean score of *M* = 1.42 and a range of [1, 2.27]. Furthermore, we divided the sentences into four groups via the Latin method and recruited 48 participants to assess the plausibility of each preview word embedded in each sentence frame on a 5-point scale (from 1 = not at all plausible to 5 = very plausible). The average plausibilities are shown in Table 1. There was no significant difference in plausibility between IP and SP (*t*(158) = 0.57, *p* = 0.57) or between TP and CP (*t*(158) = 0.89, *p* = 0.38). The TP and CP conditions were also balanced in terms of character structure, word frequency (Cai & Brysbaert, 2010) (*t*(158) = 0.03, *p* = 0.97), word complexity (*t*(158) = 0.22, *p* = 0.83), frequency of the left character (*t*(158) = 1.38, *p* = 0.17), frequency of the right character (*t*(158) = 1.81, *p* = 0.07), the complexity of the left character (*t*(158) = 0.06, *p* = 0.95), and the complexity of the right character (*t*(158) = 0.17, *p* = 0.86).

### 2.4. Material in Experiment 2

Experiment 2 supplemented Experiment 1 by employing preview words whose transposed forms did not constitute valid words. For example, the transposed–semantic preview word 饱满 was rearranged into 满饱, which is not a valid word, as illustrated in Figure 2. The experimental materials were adapted from Zhu et al. (2021), where the preview words were either synonyms or semantically related words of the target words. Synonyms were selected as semantically related previews. If the transposed form of the synonym constituted a valid word, semantically related words were used instead. As a result, both the transposed previews and control previews were nonwords. To ensure comparability between conditions, we verified no significant differences in linguistic properties between the transposed and control preview conditions. Specifically, thirty-eight participants evaluated the plausibility of each preview stimuli embedded in each sentence frame on a 5-point scale (from 1 = not at all plausible to 5 = very plausible). The results showed that the SP (*M* = 3.55, *SD* = 0.58) condition was more plausible than the TP (*M* = 2.76, *SD* = 0.50) condition, *t* = 9.19, *p* < 0.001. The TP condition was more plausible than the CP (*M* = 2.10, *SD* = 0.34) condition, *t* = 9.75, *p* < 0.001. In addition, there were no significant differences in the first character frequency (*t*(158) = 0.23, *p* = 0.82), last character frequency (*t*(158) = 0.55, *p* = 0.58), first character stroke count (*t*(158) = 0.02, *p* = 0.98), last character stroke count (*t*(158) = 0.56, *p* = 0.58), and overall stroke count (*t*(158) = 0.37, *p* = 0.71) between the matched transposed preview and control preview conditions, as shown in Table 1.

We adopted a counterbalanced design in which the experimental sentences were divided into four lists, and one version of each sentence frame was in one list. Each participant was pseudorandomly allocated to one list to ensure the equal distribution of participants across lists and an equal number of sentences in each condition. Each list also included 40 filler sentences and began with six practice sentences. The filler and experimental sentences were intermixed and presented in random order.

### 2.5. Apparatus and Procedure

An SR Eyelink 1000 eye tracker tracked right-eye movements during binocular viewing at a 1000 Hz sample rate. The stimuli were presented on a 24-inch LCD monitor with a resolution of (1920 × 1080 pixels) and a refresh rate of 165 Hz. Sentences were displayed in Song 36-point font in black (RGB: 0, 0, 0) on a grey background (RGB: 128, 128, 128). At a 60 cm viewing distance, each character subtended 1.2° and so was of normal size for reading. Participants read the sentences binocularly, but only the right eye was monitored.

Participants took part individually and were instructed to read normally in a self-paced manner and for comprehension. At the start of the experiment, a 3-point horizontal calibration procedure was performed across the same line as each sentence presentation (ensuring 0.3°or better spatial accuracy for all participants). Calibration accuracy was checked before each trial, and the eye tracker was recalibrated as required to maintain high spatial accuracy. At the start of each trial, a cross equal in size to one character was presented on the left side of the screen. Once the participant fixated on this location, the first half-sentence was presented with the first character replacing the cross. Participants pressed the space key once they finished reading the sentence. This was replaced by a comprehension question requiring a yes/no button-press response (with “yes” indicated by the “F” key and “no” by the “J” key) in 25% of trials. The experiment lasted approximately 30 min for each participant.

### 2.6. Data Analysis

Accuracy in answering comprehension questions was high across all participants (Experiment 1: *M* = 94%, *SD* = 5%, range [75%, 100%]; Experiment 2: *M* = 97%, *SD* = 4%, range [85%, 100%]). Following standard processing procedures, short (<80 ms) and long (>1200 ms) fixations were excluded, which affected 7% of fixations in Experiment 1 and 8% of fixations in Experiment 2. Trials interrupted by extraneous events (e.g., sneezing, coughing) were also excluded, resulting in the removal of 2 trials in Experiment 1 and 1 trial in Experiment 2. Additionally, trials with fewer than five fixations per sentence were removed (242, 5.04% trials in Experiment 1; 135, 2.81% in Experiment 2). Further exclusions were made for trials in which an eye-blink occurred within the boundary region or while fixating the target word (affecting 39 trials, 0.8% in Experiment 1; and 23 trials, 0.48% in Experiment 2), as well as for trials in which a saccade triggered a display change but terminated to the left of the target word (i.e., a “j-hook”, affecting 153 trials, 3.19% in Experiment 1; 185 trials, 3.85% in Experiment 2). Finally, trials in which display changes were delayed by more than 10 ms into the subsequent fixation were excluded (990 trials, 20.6% in Experiment 1; 1025 trials, 21.35% in Experiment 2). In total, 1114 trials (23.21%) in Experiment 1 and 1199 trials (24.98%) in Experiment 2 were removed from analysis. Participants who reported noticing display changes in more than 10% of trials were excluded (7 participants in Experiment 1 and 8 participants in Experiment 2, who were replaced), aligning with criteria used in prior research (Chang et al., 2025; He et al., 2021). The final sample consisted of 60 valid participants for each experiment.

The remaining data were analyzed by linear mixed-effects models (Baayen et al., 2008) for continuous variables and generalized mixed-effects models for binomial variables, using the lme4 package (Bates et al., 2015) in R (R Development Core Team, 2016). For all measures, models with the maximum random-effects structure were used (Barr et al., 2013), with the preview condition as a fixed factor and participants and stimuli as crossed random effects. If models did not converge, the random-effects structure was reduced by first trimming this for stimuli. Contrasts were defined using the contr.sdif function in the MASS package (Version 7.3-60; Venables & Ripley, 2002). Following convention, |*t*/*z*|s values > 1.96 were considered significant.

For the two experiments, we reported seven measures for the target words, i.e., word-skipping (SKIP, probability of not fixating a word during first-pass reading), first-fixation duration (FFD, duration of the first fixation on a word during first-pass reading), single-fixation duration (SFD, duration of the first fixation on a word receiving only one first-pass fixation), and gaze duration (GD, sum of all first-pass fixations on a word). These four early eye movement measures are sensitive to pre-activation and the lexical access of target words during the parafoveal preview phase, making them ideal for capturing the immediate effects of character transposition, aligning with prior studies in the field (e.g., Chang et al., 2025; Gu et al., 2015). We also reported two measures concerning later semantic integration (see Supplement Tables S1 and S2), i.e., regression path duration (RPD, the sum of all fixation durations beginning with the initial fixation on the target word and ending when the eyes exited the word to the right, including time spent rereading earlier words and time spent rereading the word itself) and total reading time (TRT, sum of all fixations on a target word).

## 3. Results

**Figure 3:**
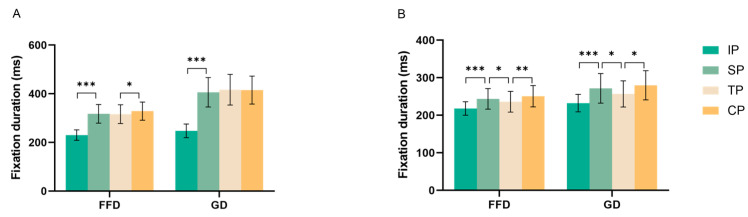
Fixation durations for the four experimental conditions in Experiment 1 (Panel **A**) and Experiment 2 (Panel **B**). * *p* < 0.05, ** *p* < 0.01, *** *p* < 0.001.

The descriptive statistical results of target words are shown in Table 2, and statistical effects are summarized in Table 3, inter-condition contrasts are visually depicted in Figure 3.

### 3.1. Experiment 1: Results and Discussion

The comparison of preview benefits between the two forms of reversible words (i.e., semantic preview vs. transposed–semantic preview) revealed no significant differences, e.g., the preview benefits for the target word 西装 (tie) were similar when previewing领带 (tie) and 带领 (lead), as indicated by comparable measures across all measures (|*t*/*z*|s ≤ 1.08). This suggests that when readers preview the meaning of 带领 (lead), the meaning of 领带 (tie) is also activated, which similarly facilitates the processing of the target 西装 (suit).

The transposed form (带领_lead) of semantic preview stimuli produced significant preview benefit compared to the invalid controlled preview (TP vs. CP) across three early eye movement measures: SKIP (*b* = −0.24, CI = [−0.45, −0.03], *SE* = 0.11, *z* = −2.22), FFD (*b* = 16.16, CI = [2.33, 30], *SE* = 7.06, *t* = 2.29), and SFD (*b* = 29.88, CI = [13, 47], *SE* = 8.69, *t* = 3.44). After controlling for semantic relatedness and plausibility, the processing advantage for TP over CP remained significant for FFD (*b* = 19, *SE* = 9, *t* = 2.28) and SFD (*b* = 28, *SE* = 10, *t* = 2.68). However, no significant difference emerged between TP and CP regarding GD. The reason might be that the TP word (e.g., 带领) forms a valid word with distinct semantic meaning (“lead”), which might compete with the target word’s semantics (e.g., “suit”). This semantic conflict prolongs GD as the visual system resolves the competition between preview and target meanings, resulting in no significant GD difference between TP and CP.

These results demonstrate that the transposed–semantic preview condition (TP) facilitated word processing similarly to the semantic preview condition (SP) and provided a processing advantage for the TP condition over the controlled preview condition (CP). Such findings can be attributed to flexible character position encoding during semantic preview processing. This supports the hypothesis that character order encoding is flexible during the initial stages of word processing, which may coincide with semantic preview processing in the parafoveal region.

While Experiment 1 employed reversible words as preview stimuli, it is possible that the semantic information provided by the transposed-preview (TP) condition interfered with the processing of the target word. This raises the question of whether a transposed–semantic preview benefit would still be observed when using non-reversible words as preview stimuli. Experiment 2 will address this issue by using non-reversible words to determine whether the transposed–semantic preview benefit is preserved under these conditions.

### 3.2. Experiment 2: Results and Discussion

The comparison between semantic preview (SP) and transposed–semantic preview (TP) revealed a processing advantage for TP over SP in both FFD and GD (FFD: *b* = −10, CI = [−19, 0], *SE* = 4.77, *t* = −2.03; GD: *b* = −16, CI = [−30, −2], *SE* = 7.02, *t* = −2.28). Consistent with the results of Experiment 1, there was no significant difference between TP and SP in skipping rate, SFD, indicating comparable processing for both semantic and transposed–semantic preview conditions.

The comparison between the transposed–semantic preview (TP) and control condition (CP) demonstrated a significant processing advantage for TP over the CP condition in FFD (*b* = 13, CI = [4, 22], *SE* = 4.71, *t* = 2.73), SFD (*b* = 11, CI = [2, 21], *SE* = 4.93, *t* = 2.29), and GD (*b* = 17, CI = [4, 31], *SE* = 6.93, *t* = 2.51). After controlling for sentence plausibility, the TP > CP advantage remained significant for FFD (*b* = 11, *SE* = 6, *t* = 1.96) and SFD (*b* = 11, *SE* = 6, *t* = 1.92), consistent with the results of Experiment 1. This confirms that previewing transposed–semantic preview stimuli facilitate target word processing relative to the unrelated control preview stimuli. Notably, while the skipping rate was lower for the control condition than the TP condition, the difference was nonsignificant (z = −1.39). This outcome is plausibly attributed to a ceiling effect induced by the significantly higher skipping rate in Experiment 2 relative to Experiment 1, which attenuated the discriminability between the TP and control preview (CP) conditions.

Notably, Experiment 2 exhibited overall higher skipping rates and shorter fixation durations compared to Experiment 1, a discrepancy potentially rooted in material difficulty differences. Whereas Experiment 1 utilized self-constructed materials, Experiment 2 adopted stimuli from Zhu et al. (2021). Therefore, we recruited 12 participants to assess the difficulty of the sentences used in both experiments using a seven-point Likert scale. The results showed that the sentences in Experiment 2 (*M* = 2.62, *SD* = 0.34) were significantly easier than those in Experiment 1 (*M* = 2.32, *SD* = 0.39, *p* < 0.001). This difference in sentence difficulty likely contributed to the observed patterns in eye movements, as easier sentences in Experiment 2 may have promoted more efficient reading, leading to higher skipping rates and shorter fixation durations. These findings highlight the importance of considering material difficulty when interpreting cross-experimental differences in eye movement measures.

## 4. General Discussion

The present study employed the boundary paradigm across two experiments to investigate the flexibility of character position encoding during semantic preview processing in Chinese reading. Experiment 1 utilized reversible semantically related word pairs (e.g., 领带 “necktie” and 带领 “lead”), while Experiment 2 used non-reversible semantically related words (e.g., 饱满 “plump” and its transposed nonword 满饱 “manbao”) as preview stimuli. The core objective was to determine whether transposed semantic previews—regardless of whether they formed valid words—could elicit preview effects comparable to semantic previews and exhibit processing advantages over unrelated control previews, thereby providing further evidence for the flexible character order processing in the parafoveal region.

Our research engages with the ongoing debate surrounding the transposed-character effect in Chinese reading (Chang et al., 2020, 2025; Gu et al., 2015, 2023; Gu & Li, 2015; J. Yang, 2013; H. Yang et al., 2022; Zhang et al., 2022) as well as its cross-linguistic parallels in English word recognition (Blythe et al., 2014; Duñabeitia et al., 2014; Johnson et al., 2023; Kirkby et al., 2025; Luke & Christianson, 2012; Marcet et al., 2019; Massol & Grainger, 2024; Mirault & Grainger, 2021; Perea et al., 2012). The design of prior studies has typically employed transposed forms or substituted-character nonwords of target words as preview stimuli (e.g., 装西 as a transposed preview for the target 西装, 袭有 as a substituted preview for the target 西装). A consistent finding across these works is a processing advantage for transposed stimuli over unrelated substituted ones, called a transposed-character effect. This phenomenon challenges models assuming strict positional encoding (e.g., the dual-route cascaded model; Coltheart et al., 2001) and aligns with frameworks emphasizing flexible positional coding, such as the “noisy position” model (Gomez et al., 2008) and the open-bigram model (Grainger & Van Heuven, 2004).

One account for the transposed-character effect is that readers encode the character order information flexibly. This flexibility may stem from the “noisy position” of letters (Gomez et al., 2008; Whitney, 2001), whereby the orthographic code for a letter is not rigidly bound to a specific positional locus. Instead, the process of letter position coding exhibits inherent flexibility, allowing for probabilistic rather than deterministic mapping between character identities and their spatial arrangements within a word. This theoretical framework posits that positional uncertainty in orthographic representation enables partial activation of target words even when adjacent letters or characters are transposed, as the cognitive system tolerates a degree of spatial ambiguity during word recognition. In Chinese, this flexibility may be further influenced by the logographic nature of characters, where semantic and morphological cues (e.g., shared morphemes in transposed pairs) could modulate orthographic processing (H. Yang et al., 2022). Cross-linguistically, similar effects in alphabetic languages (e.g., English transposed-letter effects; for a review, see Grainger, 2024) suggest a universal mechanism for flexible positional encoding, albeit shaped by language-specific orthographic constraints (Grainger & Ziegler, 2011).

From the perspective of CRM, the transposed-character effect might arise from bottom-up activation between character and word levels (Li & Pollatsek, 2020). Even when characters are transposed, shared character identities (e.g., 装 and 西 in 西装) trigger excitatory connections to the target word, whereas substituted characters (e.g., 袭 and 有 in 袭有) lack such connections. Critically, the current study design dissociates character identity from positional flexibility by ensuring that transposed–semantic previews (e.g., 带领) and unrelated previews (e.g., 阅读) share no visual or semantic overlap with the target word (e.g., 西装). This allows us to isolate the effect of positional encoding from mere character-level activation. The results reveal two key findings:

Equivalence of semantic and transposed–semantic previews: Early eye movement measures (e.g., first fixation duration, gaze duration) show comparable processing efficiency between semantic previews (e.g., 领带) and transposed–semantic previews (e.g., 带领). This suggests that readers activate both the canonical and transposed forms of semantically related words during parafoveal processing, consistent with the finding of Peng et al. (1999).

Processing advantage of transposed–semantic previews over controls: Transposed–semantic previews (e.g., 带领) elicit shorter fixation durations than unrelated controls (e.g., 阅读), even though both are visually dissimilar to the target word. This finding cannot be explained by character-level activation alone and instead supports the existence of flexible positional encoding: readers leverage abstract positional relationships between characters, enabling semantic integration despite order disruption.

These results align with cross-linguistic evidence from alphabetic languages, where transposed-letter effects reflect a universal capacity for flexible positional coding (Grainger & Ziegler, 2011; Massol & Grainger, 2024). In Chinese, this flexibility may be further amplified by the absence of word boundaries, which necessitates robust positional tolerance for efficient sentence parsing (Li et al., 2009). Notably, the lack of a processing difference between reversible (Experiment 1) and non-reversible (Experiment 2) transposed previews suggests that lexical status (e.g., whether transposed forms are valid words) might not modulate positional flexibility. This hypothesis could be further validated by future well-controlled studies.

Our study focused on young adults. Future research should examine children and older adults to understand the lifespan development of parafoveal order processing. No studies have explored this in children. Given their smaller perceptual span (Yan et al., 2020), children may allocate more cognitive resources to the fovea, showing less flexibility in processing parafoveal Chinese character positions. Research on older adults shows that despite limited parafoveal processing (He et al., 2021), they can still flexibly handle character order in this region (Chang et al., 2025). This suggests character identity processing declines with age, while position processing remains stable. Testing these hypotheses through future studies will trace the development of character order processing across life stages, filling research gaps and enhancing the theory of word identification.

## 5. Conclusions

In summary, this study provides robust evidence for flexible character position encoding during Chinese reading, demonstrating that parafoveal processing tolerates positional disruptions in semantically related previews. The findings advance our understanding of how logographic systems balance orthographic precision with processing efficiency, shedding light on the universal and language-specific mechanisms underlying word recognition. Future research may further explore how factors like character frequency, word class, text orientation, and word validity modulate this flexibility, enhancing our grasp of reading in diverse orthographic systems, as well as how this flexibility varies across different age groups.

## Figures and Tables

**Table 1:** Stimulus characteristics of Experiment 1.

	Properties	IP		SP		TP		CP	
		L_char	R_char	L_char	R_char	L_char	R_char	L_char	R_char
Exp1	Example	西	装	领	带	带	领	阅	读
	Plausibility	3.99 (0.66)		3.93 (0.62)		2.16 (0.65)		2.06 (0.73)	
	Complexity	15.99 (3.82)		13.83 (3.92)		13.83 (3.92)		13.96 (4.02)	
	Frequency	42 (131)		36 (97)		8 (17)		8 (17)	
	Char_freq	698 (1269)	1335 (4400)	1221 (2309)	1792 (2556)	1792 (2556)	1221 (2309)	1217 (2730)	691 (1246)
	Char_comp	7.85 (2.7)	8.14 (3.28)	6.88 (2.73)	6.95 (2.81)	6.95 (2.81)	6.88 (2.73)	6.93 (2.57)	7.04 (2.79)
Exp2	Example	充	实	饱	满	满	饱	静	虾
	Plausibility	3.66 (0.5)		3.55 (0.58)		2.76 (0.5)		2.1 (0.34)	
	Complexity	17.74 (4.11)		18.16 (5.41)		18.16 (5.41)		17.85 (5.2)	
	Frequency	100 (660)		38 (95)					
	Char_freq	412 (672)	530 (1032)	1087 (3397)	564 (891)	564 (891)	1087 (3397)	601 (1104)	837 (2248)
	Char_comp	8.71 (2.7)	9.03 (3.02)	9.13 (3.61)	9.04 (3.69)	9.04 (3.69)	9.13 (3.61)	9.03 (3.42)	8.83 (3.18)

Note: The standard deviations are in parentheses. L_char and R_char represent the left and right characters, respectively. Char_freq and Char_comp represent character frequency and character complexity, respectively. IP, SP, TP, and CP refer to identical preview, semantic preview, transposed–semantic preview, and controlled preview, respectively. The preview words 西装 (suit), 领带 (tie), 带领 (leading), and 阅读 (reading) were used in Experiment 1. The preview stimuli 充实 (substantial) 饱满 (plump), the nonwords 满饱 and 静虾 were used in Experiment 2.

**Table 2:** The means and standard errors for the measures of the two experiments.

	Measures	IP	SP	TP	CP
Experiment 1	SKIP (%)	32 (11)	34 (10)	36 (11)	32 (11)
	FFD (ms)	230 (22)	318 (38)	316 (39)	329 (38)
	SFD (ms)	230 (23)	316 (51)	308 (51)	337 (49)
	GD (ms)	248 (28)	406 (60)	417 (63)	415 (57)
Experiment 2	SKIP (%)	39 (12)	38 (12)	39 (12)	35 (12)
	FFD (ms)	218 (18)	244 (27)	236 (28)	251 (28)
	SFD (ms)	218 (18)	240 (29)	233 (28)	247 (30)
	GD (ms)	232 (23)	272 (39)	257 (35)	280 (39)

Note. The standard error of the mean is shown in the parentheses. IP, SP, TP, and CP refer to identical preview, semantic preview, transposed–semantic preview, and controlled preview, respectively.

**Table 3:** Statistical effects for Experiments 1 and 2.

Measures		Estimate	CI	SE	*z*/*t*
Experiment 1					
SKIP	Intercept	−0.88	[−1.17, −0.6]	0.14	−6.26
	SP vs. IP	0.15	[−0.07, 0.36]	0.11	1.35
	TP vs. SP	0.10	[−0.11, 0.31]	0.11	0.98
	CP vs. TP	−0.24	[−0.45, −0.03]	0.11	−2.22 *
FFD (ms)	Intercept	299	[285, 313]	7.25	41.26
	SP vs. IP	90	[76, 104]	7.00	12.85 ***
	TP vs. SP	−4	[−18, 10]	7.11	−0.59
	CP vs. TP	16	[2, 30]	7.06	2.29 *
SFD (ms)	Intercept	299	[284, 314]	7.63	39.18
	SP vs. IP	88	[72, 103]	7.99	10.99 ***
	TP vs. SP	−9	[−26, 8]	8.70	−1.06
	CP vs. TP	30	[13, 47]	8.69	3.44 ***
GD (ms)	Intercept	372	[347, 397]	12.54	29.68
	SP vs. IP	164	[142, 186]	11.26	14.53 ***
	TP vs. SP	11	[−11, 34]	11.43	1.00
	CP vs. TP	−1	[−24, 21]	11.36	−0.13
Experiment 2					
SKIP	(Intercept)	−0.58	[−0.79, −0.37]	0.11	−5.45
	SP vs. IP	−0.09	[−0.29, 0.12]	0.10	−0.84
	TP vs. SP	0.07	[−0.13, 0.27]	0.10	0.68
	CP vs. TP	−0.14	[−0.35, 0.06]	0.10	−1.39
FFD (ms)	(Intercept)	236	[225, 247]	5.61	42.03
	SP vs. IP	26	[17, 36]	4.76	5.52 ***
	TP vs. SP	−10	[−19, 0]	4.77	−2.03 *
	CP vs. TP	13	[4, 22]	4.71	2.73 **
SFD (ms)	(Intercept)	234	[223, 245]	5.63	41.58
	SP vs. IP	25	[16, 35]	4.94	5.11 ***
	TP vs. SP	−9	[−19, 1]	4.98	−1.78
	CP vs. TP	11	[2, 21]	4.93	2.29 *
GD (ms)	(Intercept)	258	[241, 275]	8.45	30.54
	SP vs. IP	41	[27, 54]	7.00	5.8 ***
	TP vs. SP	−16	[−30, −2]	7.02	−2.28 *
	CP vs. TP	17	[4, 31]	6.93	2.51 *

Note: IP, SP, TP, and CP refer to identical preview, semantic preview, transposed–semantic preview, and controlled preview, respectively. * *p* < 0.05, ** *p* < 0.01, *** *p* < 0.001.

## Data Availability

The datasets and corresponding analysis code are available online at https://doi.org/10.6084/m9.figshare.28736891.v2 (accessed on 1 June 2025). Materials used in the two experiments are provided in the Supplementary Materials (Tables S3 and S4). Additionally, this study’s design, hypotheses, and analysis plans have been preregistered and can be accessed at https://os.psych.ac.cn/preregisterdetail/202401.00094. The preregister ID is 202401.00094V1 (accessed on 13 January 2024).

## Supplementary Materials

The following supporting information can be downloaded at: https://www.mdpi.com/article/10.3390/bs15070907/s1, Table S1: The means and standard errors for TRT and RPD in two experiments; Table S2: Statistical Effects in TRT and RPD for Experiments 1 and 2. Table S3: Materials in Experiment 1; Table S4: Materials in Experiment 2.
