# Supplementary material for: The Character Position Encoding of Parafoveal Semantic Previews Is Flexible in Chinese Reading
Source: Behav Sci (Basel). 2025 Jul 4;15(7):907. doi: 10.3390/bs15070907 (PMC12292683; doi:10.3390/bs15070907)
Supplement: Supplementary file 1 [file behavsci-15-00907-s001.zip › behavsci-3697661-supplementary.pdf]

Table S1. The means and standard errors for TRT and RPD in two experiments

	measures	IP	SP	TP	CP
Experiment 1	TRT (ms)	321 (47)	518 (64)	512 (73)	532 (68)
	RPD (ms)	297 (53)	503 (82)	524 (90)	554 (98)
Experiment 2	TRT (ms)	278 (39)	320 (51)	319 (50)	329 (49)
	RPD (ms)	263 (41)	317 (58)	300 (54)	342 (65)

Note. The standard error of the mean is shown in the parentheses. IP, SP, TP, and CP refer to Identical Preview, Semantic Preview, Transposed-semantic Preview, and Controlled Preview, respectively.

Table S2. Statistical Effects in TRT and RPD for Experiments 1 and 2

measures		*Estimate*	*CI*	*SE*	*t*
Experiment 1					
TRT (ms)	Intercept	469.66	[429, 510]	20.36	23.07
	**condition2-1**	**210.22**	**[183, 237]**	**13.84**	**15.19*** **
	condition3-2	-10.37	[-37, 16]	13.45	-0.77
	condition4-3	21.89	[-4, 48]	13.32	1.64
RPD (ms)	Intercept	468.97	[427, 511]	21.31	22
	**condition2-1**	**214.98**	**[182, 248]**	**16.81**	**12.79*** **
	condition3-2	18.46	[-15, 52]	17.07	1.08
	**condition4-3**	**34.07**	**[1, 67]**	**16.97**	**2.01* **
Experiment 2					
TRT (ms)	(Intercept)	308	[287, 327]	10.16	30.27
	**condition2-1**	**46**	**[28, 65]**	**9.4**	**4.92*** **
	condition3-2	-4	[-22, 15]	9.35	-0.39
	condition4-3	6	[-13, 24]	9.33	0.61
RPD (ms)	(Intercept)	304	[281, 327]	11.68	26.07
	**condition2-1**	**55**	**[31, 79]**	**12.02**	**4.58*** **
	condition3-2	-20	[-44, 3]	12.06	-1.69
	**condition4-3**	**37**	**[14, 60]**	**11.92**	**3.11** **

Note: * denotes statistical significance (p<0.05). Condition 2-1 presents the comparison between semantic preview (SP) and valid identical preview (IP). Condition 3-2

compares the transposed-semantic preview (TP) with the SP condition, while Condition 4-3 highlights the processing advantage of TP relative to the unrelated controlled condition (CP).

Table S3. Materials in Experiment 1

Item	Sentence
1	这家奢侈品牌卖的蓝色 [西装/领带/带领/阅读] 非常受消费者欢迎。
1	The blue [suit/tie/misleading/reading] sold by this luxury brand is very popular among consumers.
2	农民工为国家的建设贡献了 [全部/所有/有所/不到] 的智慧和力量。
2	Migrant workers have contributed [all/every/partially/insufficient] wisdom and strength to the country's construction.
3	刘慈欣写的科幻 [小说/故事/事故/团队] 把我们带进了一个奇妙的世界。
3	The sci-fi [novel/story/accident/team] written by Liu Cixin has brought us into a wonderful world.
4	这次野营拉练活动暴露出 [个别/部分/分部/尺码] 同学怕吃苦的弱点。
4	The camping training activity has revealed that [individual/some/department/size] students are afraid of hardship.
5	李铭用大量的 [证据/事实/实事/全票] 有力地驳斥了对手的恶毒攻击。
5	Li Ming forcefully refuted the opponent's vicious attack with a large amount of [evidence/facts/real issues/unanimous votes].
6	刘科长的言论在群众中造成了 [消极/不好/好不/现今] 的影响和后果。
6	Section Chief Liu's remarks have caused [negative/bad/extremely/current] impacts and consequences among the masses.
7	这几个作恶多端的 [凶手/罪犯/犯罪/投票] 终于被警方缉拿归案了。
7	These heinous [murderers/criminals/crimes/votes] have finally been arrested by the police.
8	长期处在失去 [女儿/家人/人家/出口] 的哀痛之中使得这位母亲精神失常。
8	Being in the grief of losing her [daughter/family/people/export] for a long time has made this mother mentally deranged.
9	电影节红毯上这位 [妩媚/性感/感性/费劲] 的女明星吸引了很多人的注意。
9	This [charming/sexy/sensitive/laborious] female star on the film festival red carpet has attracted a lot of attention.
10	爸爸决定明天下午就 [启程/出发/发出/生存] 回湖南老家看望奶奶。
10	Dad decided to [set out/depart/send/survive] tomorrow afternoon to return to his hometown in Hunan to visit grandma.
11	犯罪分子们把 [炸药/子弹/弹子/格局] 和抢劫得来的赃物都藏在了这辆火车里。
11	The criminals hid [dynamite/bullets/marbles/pattern] and the stolen goods from robbery in this train.
12	我国京剧表演艺术家在 [欧洲/法国/国法/甲级] 的演出引起了巨大轰动。
12	The performances of Chinese Peking Opera artists in [Europe/France/national law/Class A] caused a great sensation.
13	李明明因为生病住院而 [耽误/错过/过错/进化] 了半学期的课程。
13	Li Mingming [delayed/missed/fault/evolved] half a semester's courses because of being hospitalized.
14	高老师对 一些 [国家/社会/会社/名酒] 丑恶现象的抨击往往一针见血。
14	Teacher Gao's criticism of the ugly phenomena in some [countries/society/company/famous wines] is often to the point.

15 这幅油画描绘了一位中世纪的 [骑士/国王/王国/毛衣] 拼命斩杀恶龙的场景。
15 This oil painting depicts a medieval [knight/king/kingdom/sweater] desperately slaying a dragon.
16 红外线摄影技术可以捕捉到遥远 [宇宙/星球/球星/船尾] 的令人震撼的全彩照片。
16 Infrared photography technology can capture stunning full-color photos of distant [universes/planets/star players/ship tails].
17 陆教授近年来致力于 [理论/科学/学科/货舱] 研究并积极进行跨学科的探索。
17 Professor Lu has been dedicated to [theoretical/scientific/disciplinary/cargo hold] research in recent years and actively carried out interdisciplinary exploration.
18 人类科技的不断进步使得 [完成/实现/现实/将军] 太空旅行的梦想近在咫尺。
18 The continuous progress of human science and technology has made the dream of [completing/realizing/reality/general] space travel close at hand.
19 孩子们坐在车上好奇地看着[路边/街上/上街/出境] 川流不息的人群。
19 The children sat in the car and curiously looked at the endless stream of people on the [roadside/street/go shopping/exit].
20 这位漂亮阿姨的实际 [岁数/年纪/纪年/烟雨] 比外表看起来要大许多。
20 The actual [age/years/calendar/misty rain] of this beautiful aunt is much older than she looks.
21 心胸狭隘的 [王后/公主/主公/头巾] 总是嫉妒那位美丽的公爵小姐。
21 The narrow-minded [queen/princess/duke/headscarf] is always jealous of the beautiful duke's daughter.
22 加大投资力度是未来三年庐江生产 [总部/基地/地基/插座] 的扩产目标。
22 Increasing investment is the production expansion goal of Lujiang production [headquarters/base/foundation/socket] in the next three years.
23 这段文字对主人公 [外貌/形象/象形/留待] 的描写十分具体概括。
23 This paragraph describes the protagonist's [appearance/image/pictograph/remain] in a very specific and comprehensive way.
24 内心阴暗的 [爱人/情人/人情/小便] 显然比素不相识的陌生人还危险。
24 A dark-hearted [lover/lover/human feeling/urination] is obviously more dangerous than a stranger.
25 这部小说成功地塑造了一个 [吝啬/自私/私自/抢先] 商人的丑恶形象。
25 This novel has successfully created an ugly image of a [stingy/selfish/private/preemptive] merchant.
26 这个国际大毒枭的 [同伙/手下/下手/当心] 已经被警察一网打尽。
26 The [accomplices/subordinates/start/careful] of this international drug lord have been completely arrested by the police.
27 林则徐看到那台用 [白银/黄金/金黄/无度] 从欧洲买来的哑炮怒火中烧。
27 Lin Zexu was furious when he saw the dud bought from Europe with [silver/gold/golden/excessive] money.
28 那只宠物狗在太阳下 [伸展/张开/开张/来往] 了四肢并奔向他的主人。
28 The pet dog [stretched/opened/opened/come and go] its limbs in the sun and ran towards its owner.
29 李明浩终于如愿考入了 [北京/上海/海上/脸色] 的一所著名大学。

29 Li Minghao finally succeeded in being admitted to a famous university in [Beijing/Shanghai/sea/face].

30 那位被紧急送到 [医院/产房/房产/处方] 的孕妇平安地生下了孩子。

30 The pregnant woman who was urgently sent to the [hospital/maternity ward/real estate/prescription] gave birth to a child safely.

31 这家食品企业生产出来的 [饮料/汽水/水汽/石阶] 非常受大众喜爱。

31 The [beverages/sodas/water vapor/stone steps] produced by this food enterprise are very popular with the public.

32 她在市政府 [单位/机关/关机/受损] 的工作中展现了卓越的领导才能。

32 She has demonstrated excellent leadership skills in her work in the municipal government [unit/authority/power off/damaged].

33 警察们迅速使用 [武器/手枪/枪手/相片] 阻止了犯罪分子的逃离。

33 The police quickly used [weapons/pistols/gunmen/photos] to prevent the criminals from escaping.

34 一个人取得成功的 [条件/前提/提前/依然] 是他有能力改变自己并适应环境。

34 The [condition/prerequisite/advance/still] for a person to achieve success is that he has the ability to change himself and adapt to the environment.

35 刘教授常常通过 [邮件/传真/真传/拳师] 发送重要的科研文件和资料。

35 Professor Liu often sends important scientific research documents and materials via [email/fax/true biography/boxer].

36 公司总部的 [宣传/公关/关公/古田] 部门紧急召开了新闻发布会。

36 The [propaganda/public relations/Guan Gong/Gu Tian] department of the company headquarters urgently convened a press conference.

37 住了两年多的隔壁 [租户/房客/客房/宿舍] 在大兴买了套房后就立马搬走了。

37 The next-door [tenant/roomer/guest room/dormitory] who had lived there for more than two years moved out immediately after buying an apartment in Daxing.

38 她非常厌倦贵族的那种荒淫 [无耻/下流/流下/折中] 的豪门生活。

38 She is very tired of the licentious [shameless/obscene/flow/eclectic] life of the noble family.

39 专家认为每天早晨喝 [豆浆/牛奶/奶牛/外衣] 吃鸡蛋有益于身体健康。

39 Experts believe that drinking [soybean milk/milk/cow/coat] and eating eggs every morning is beneficial to health.

40 从上海开往 [西藏/拉萨/萨拉/前科] 的火车将经过世界上海拔最高的铁路线。

40 The train from Shanghai to [Tibet/Lhasa/Sara/criminal record] will pass through the railway line with the highest altitude in the world.

41 莉莉的英国男朋友已经将 [汉语/中文/文中/出工] 说的非常流利了。

41 Lily's British boyfriend has spoken [Chinese/Chinese/literary/going to work] very fluently.

42 小区物业的工作人员对 [住户/业主/主业/广角] 的投诉总是应付了事。

42 The staff of the community property always perfunctorily deal with the complaints from [residents/property owners/main business/wide-angle].

43 我们单位的 [保安/门卫/卫门/成虫] 大叔们工作态度非常认真负责。

43 The [security guards/doormen/Wei Men/adults] in our unit have a very serious and responsible work attitude.

44 这次的医疗腐败案是导致总统 [卸任/下台/台下/合乎] 的主要因素。

44 This medical corruption case was the main factor leading to the president's [resignation/downfall/under the stage/conformity].
45 深入理解圆的 [周长/直径/径直/判定] 这一概念有助于解决许多实际问题。
45 Deeply understanding the concept of the [circumference/diameter/straight/judgment] of a circle helps solve many practical problems.
46 李明利用暑假特意去 [青岛/山东/东山/凡世] 看望了大学时的班主任张老师。
46 Li Ming specially went to [Qingdao/Shandong/East Mountain/every world] during the summer vacation to visit his university head teacher, Teacher Zhang.
47 国家提出发展农村经济的 [目的/动机/机动/阴险] 是改善农民生活。
47 The [purpose/motivation/mobile/sinister] of the country's proposal to develop the rural economy is to improve the lives of farmers.
48 父母的一言一行都会对 [孩子/子女/女子/本人] 产生潜移默化的影响。
48 Every word and deed of parents will have a subtle influence on their [children/children/daughters/oneself].
49 晓玲下定决心要 [努力/用功/功用/蚊虫] 学习以考上理想的学校。
49 Xiaoling is determined to [study hard/work hard/function/mosquito] to enter the ideal school.
50 妈妈叮嘱明明将那个 [腐烂/变质/质变/居多] 的苹果扔进垃圾桶里。
50 Mom told Mingming to throw the [rotten/spoiled/qualitative change/most] apple into the trash can.
51 英语老师明确地指出了琳琳 [发音/语法/法语/楼梯] 中的三个错误。
51 The English teacher clearly pointed out three mistakes in Linlin's [pronunciation/grammar/French/stairs].
52 刘老师将自己所有的 [财产/家当/当家/下蛋] 都交托给了新婚的爱人。
52 Teacher Liu entrusted all his [property/possessions/head of the family/lay eggs] to his newlywed lover.
53 在我们对面的 [河边/岸上/上岸/出息] 是一片黄澄澄的燕麦田。
53 Opposite us, on the [riverbank/shore/go ashore/prospects] is a yellow oat field.
54 几块具有重大意义的 [标本/化石/石化/上浮] 皆出自业余挖掘者的手中。
54 Several significant [specimens/fossils/petrifaction/float] all came from the hands of amateur excavators.
55 《悲惨世界》这部外国 [经典/名著/著名/复杂] 我已经通读过两遍了。
55 I have read the foreign [classic/masterpiece/famous/complex] "Les Misérables" twice.
56 新来的同事费力 [讨好/巴结/结巴/神龙] 领导的行为让人非常地鄙视。
56 The new colleague's behavior of trying hard to [please/flatter/stutter/dragon] the leader is very contemptible.
57 屠呦呦因在治疗疟疾方面的 [杰出/伟大/大伟/见地] 成就而备受世人尊敬。
57 Tu Youyou is highly respected by the world for her [outstanding/great/Dawei/insight] achievements in the treatment of malaria.
58 梁朝伟在这部电影里饰演的是一位 [多情/花心/心花/四声] 的小说作家。
58 Tony Leung plays a [amorous/playboy/heart flower/four tones] novel writer in this movie.
59 公司总部正在计划 [辞退/开除/除开/位居] 一批业务能力不达标的员工。
59 The company headquarters is planning to [dismiss/fire/remove/live] a group of employees with substandard business capabilities.

60 耶鲁大学要求所有的教授均讲授 [硕士/本科/科本/驮马] 课程并指导论文。
60 Yale University requires all professors to teach [master's/undergraduate/undergraduate/pack horse] courses and supervise theses.
61 王老师对于过去 [学者/名人/人名/开水] 的学说经常持怀疑的态度。
61 Teacher Wang often holds a skeptical attitude towards the theories of past [scholars/celebrities/person names/boiled water].
62 著名摄影师指出这张照片的 [明暗/色调/调色/服毒] 有很大的问题。
62 The famous photographer pointed out that the [light and shade/tone/color adjustment/poison] of this photo has serious problems.
63 早晨起床后喝一杯 [果汁/温水/水温/本镇] 对于保持皮肤健康非常有益。
63 Drinking a glass of [juice/warm water/water temperature/our town] after getting up in the morning is highly beneficial for maintaining skin health.
64 妈妈让红红把桌上的 [盘子/饭盒/盒饭/军规] 洗干净放回碗柜里。
64 Mom asked Honghong to wash the [plates/lunch boxes/bento boxes/military regulations] on the table and put them back in the cupboard.
65 大部分植物是当地国营农场的 [暖房/温室/室温/罪状] 里培育出来的。
65 Most plants were cultivated in the [greenhouse/greenhouse/room temperature/crime] of the local state-owned farm.
66 鹏鹏拿着爸爸给他买的 [彩铅/画笔/笔画/臭氧] 画了一幅日出图。
66 Pengpeng used the [colored pencils/paintbrushes/strokes/ozone] bought by his father to draw a sunrise.
67 这部电影讲述了主人公在 [随从/部下/下部/丑话] 的陪伴下回到祖国的故事。
67 The movie tells the story of the protagonist returning to his motherland accompanied by [attendants/subordinates/lower part/ugly words].
68 我在家发现一条 [蜈蚣/毒蛇/蛇毒/烟幕] 后担惊受怕地过了一夜。
68 After finding a [centipede/poisonous snake/snake venom/smoke screen] at home, I spent a night in fear.
69 李鹏和同事们经常把打 [扑克/台球/球台/洋芋] 作为闲暇时的一种消遣。
69 Li Peng and his colleagues often take [poker/billiards/pool table/potato] as a pastime in their spare time.
70 爸爸这个月的工资除去家里的 [开销/房租/租房/海鸟] 还剩余很多。
70 After deducting the family [expenses/rent/renting/sea birds] from this month's salary, Dad still has a lot left.
71 阳阳只要吃到香软的 [奶酪/牛乳/乳牛/肘子] 蛋糕就会开心的不得了。
71 Yangyang will be extremely happy as long as he eats soft and fragrant [cheese/cow's milk/dairy cow/elbow] cake.
72 古往今来的 [墨客/文人/人文/才女] 写下的诗词是我们宝贵的文化遗产。
72 Poems written by [scholars/literati/humanities/talented women] throughout the ages are our precious cultural heritage.
73 我们班主任总是以 [温柔/亲和/和亲/礼花] 的态度对待所有的学生。
73 Our head teacher always treats all students with a [gentle/affable/peaceful/fireworks] attitude.
74 王建国因为一时 [胆怯/心虚/虚心/适于] 而错失了这次晋升的机会。

74 Wang Jianguo missed the promotion opportunity due to momentary [cowardice/guilt/modesty/suitability].
75 这位数学天才对于所获得的 [荣誉/奖金/金奖/青鱼] 从未显露出得意之情。
75 This mathematical genius has never shown complacency about the [honors/bonuses/gold medals/black carp] he has received.
76 这座沿江小城已经崛起为一个 [和谐/文明/明文/祖业] 富强的现代化城市。
76 This small riverside city has risen into a [harmonious/civilized/explicit text/ancestral business] and prosperous modern city.
77 奶奶炖了许多美味的红烧 [排骨/牛肉/肉牛/义父] 为孩子们增强体力。
77 Grandma stewed a lot of delicious braised [spare ribs/beef/beef cattle/adoptive father] to strengthen the children's physique.
78 孤儿院里孩子们床上破旧的 [毛毯/被单/单被/复试] 已经难辨原貌。
78 The tattered [blankets/bed sheets/single quilts/reexamination] on the children's beds in the orphanage have become unrecognizable.
79 这家服装厂专注于销售比 [商场/市面/面市/忽闪] 价格更为经济实惠的羽绒服。
79 This garment factory specializes in selling down jackets at prices more affordable than [shopping malls/market/launch/flicker].
80 隔壁的刘奶奶总是以亲切 [和善/友好/好友/他家] 的态度对待周围的人。
80 Grandma Liu next door always treats people around her with a [kind and friendly/friendly/good friend/his family] attitude.

Note. For instance, in Item 1, the four preview conditions are [西装/领带/带领/阅读], respectively.

Table S4. Materials in Experiment 2

Item	Sentence
1	科学的生活方式是给自己安排 [充实/饱满/满饱/静虾] 的计划并实施。
1	A scientific way of life is to make [fulfilling/plump/满饱/静虾] plans for yourself and implement them.
2	妈妈生病时他尽心尽力 [服侍/伺候/候伺/哪沸] 并一直陪伴在她的身边。
2	When his mother was ill, he devoted himself to [serving/attending/候伺/哪沸] and always stayed by her side.
3	陈胜吴广起义 [推翻/颠覆/覆颠/繁膜] 了秦始皇想要百世千世的美梦。
3	The Chen Sheng-Wu Guang Uprising [overthrew/subverted/覆颠/繁膜] the dream of the First Emperor of Qin to rule for generations.
4	中国的历代奸臣都善于 [恭维/奉承/承奉/妻京] 皇帝以达到私人目的。
4	Treacherous officials in Chinese history were good at [flattering/toadying/承奉/妻京] the emperor to achieve private ends.
5	他在警察局向警官仔细 [阐明/解释/释解/疑新] 并描绘了事实的真相。
5	At the police station, he carefully [clarified/explained/释解/疑新] and described the truth to the police officer.
6	被伤害过的人想要将往事 [勾销/抹杀/杀抹/全沿] 是很难或者不能做到的。
6	It is difficult or impossible for those who have been hurt to [write off/erase/杀抹/全沿] the past.
7	作为一个真正 [杰出/卓越/越卓/超姜] 的人要德智体美劳全方面发展。
7	As a truly [outstanding/excellent/越卓/超姜] person, one should develop morally, intellectually, physically, aesthetically and laboriously.
8	没有人会无条件原谅甚至忘记 [损害/摧残/残摧/河腔] 自己利益的事。
8	No one will unconditionally forgive or even forget things that [damage/devastate/残摧/河腔] their own interests.
9	他因为无意中受到父亲的一顿 [申斥/责怪/怪责/哈某] 而心里特别难受。
9	He felt very uncomfortable because he was inadvertently [reprimanded/blamed/怪责/哈某] by his father.
10	这帮专横的政客 [把持/控制/制控/待唱] 着舆论工具以颠倒黑白迷惑群众。
10	These overbearing politicians [hold/control/制控/待唱] over public opinion tools to confuse the masses by reversing black and white.
11	这个嫌疑犯的虚伪 [原形/本相/相本/放尔] 在睿智的警官面前很快就暴露了。
11	The suspect's hypocritical [true colors/original appearance/相本/放尔] was quickly exposed in front of the wise police officer.
12	他在众人的一片 [赞扬/称颂/颂称/耿勒] 声中始终保持着冷静的头脑。
12	He always kept a cool head in the midst of [praise/eulogy/颂称/耿勒] from the crowd.
13	许多学者将自己的年轻 [身躯/肉体/体肉/抱州] 奉献给了科学研究事业。
13	Many scholars dedicated their young [bodies/flesh/体肉/抱州] to the cause of scientific research.
14	那些他们一直 [争执/辩论/论辩/担傲] 的问题始终没有得到最终的答案。
14	Those problems they have been [arguing/debating/论辩/担傲] about have never been finally answered.

15 许多条饥饿的鳄鱼 [残忍/暴虐/虐暴/盾登] 地撕扯着奄奄一息的猎物。
Many hungry crocodiles tear at the dying prey [cruelly/savagely/虐暴/盾登].
16 地震单位根据种种现象 [推测/预计/计预/任值] 出地震来临的具体日期。
16 Seismic units [inferred/predicted/计预/任值] the specific date of the earthquake based on various phenomena.
17 这个厚颜无耻的骗子利用 [怜悯/同情/情同/错友] 之心来骗取大量的钱财。
17 This brazen liar used [pity/compassion/情同/错友] to defraud a large amount of money.
18 精密仪器制作的细小 [差异/不同/同不/友么] 能导致研究结果的不准确。
18 Small [differences/differences/同不/友么] in the manufacture of precision instruments can lead to inaccurate research results.
19 伊拉克的人民总是 [向往/憧憬/憬憧/臆朦] 和平能降临他们这个国家。
19 The people of Iraq always [yearn for/long for/憬憧/臆朦] for peace to come to their country.
20 家里由于爸爸的退休也变得 [沉寂/安静/静安/满学] 起来而门可罗雀了。
20 With his father's retirement, the family has become [quiet/peaceful/静安/满学] and few visitors come.
21 经验丰富的警官由他心慌 [胆怯/害怕/怕害/护笑] 的表现来确定他的嫌疑。
21 Experienced police officers determined his suspicion from his flustered [cowardice/fear/怕害/护笑] behavior.
22 一群迁徙中的候鸟在天空 [破晓/黎明/明黎/诉裂] 时分飞过高耸的树梢。
22 A group of migrating birds flew over the tall treetops at [dawn/daybreak/明黎/诉裂].
23 百花因为在夏天吐露 [芬芳/馥郁/郁馥/鳃拆] 而吸引了路边的行人停下脚步。
23 Flowers attract passers-by to stop by pouring out [fragrance/aromatic/郁馥/鳃拆] in summer.
24 任性的同学 [索性/干脆/脆干/桶己] 将废弃的垃圾扔在不起眼的角落。
24 The willful classmate [simply/directly/脆干/桶己] threw the waste into an inconspicuous corner.
25 有专家指出地面土质 [疏松/分散/散分/搜全] 为形成沙尘暴提供了条件。
25 Experts point out that the loose [soil/dispersion/散分/搜全] on the ground provides conditions for the formation of sandstorms.
26 他将自己所有的积蓄用来 [安葬/掩埋/埋掩/浴援] 因车祸而身亡的妻子。
26 He used all his savings to [bury/inter/埋掩/浴援] his wife who died in a car accident.
27 我们不能再因为放任自己 [懒惰/怠慢/慢怠/博栗] 而丧失了自主动手能力。
27 We can no longer lose our ability to take the initiative because we indulge our [laziness/neglect/慢怠/博栗].
28 她考虑到我的再三请求终于 [答应/同意/意同/家友] 了带我去迪士尼乐园。
28 Considering my repeated requests, she finally [agreed/consented/意同/家友] to take me to Disneyland.
29 有效使用防晒产品可以 [保护/捍卫/卫捍/牛娩] 肌肤不受紫外线的伤害。
29 The effective use of sunscreen products can [protect/defend/卫捍/牛娩] the skin from UV damage.
30 科学家们有时可以通过大胆 [猜测/推想/想推/要娜] 从而提出了合理的假设。
30 Scientists can sometimes put forward reasonable hypotheses through bold [guessing/reasoning/想推/要娜].
31 人类历史文明是没有 [国界/边境/境边/搭处] 并且属于全人类的财富。

31 Human historical civilization has no [borders/frontiers/境边/搭处] and belongs to the wealth of all mankind.

32 我依稀记得昨天我正在 [洗澡/沐浴/浴沐/洋枫] 的时候手机铃响了起来。

32 I vaguely remember that my mobile phone rang when I was [bathing/showering/浴沐/洋枫] yesterday.

33 毫无头绪的他决定离开这个 [吵闹/喧嚣/嚣喧/薰惰] 的环境来集中注意。

33 He had no clue and decided to leave this [noisy/bustling/嚣喧/薰惰] environment to concentrate.

34 有些人通过在网络上散布 [流言/蜚语/语蜚/油蓉] 的方式来获取关注度。

34 Some people spread [gossip/slander/语蜚/油蓉] on the Internet to gain attention.

35 我们必须明白只有在经过一番 [波折/坎坷/坷坎/柚扩] 后才能真正取得成功。

35 We must understand that only after going through some [twists and turns/ups and downs/坷坎/柚扩] can we truly succeed.

36 我们不仅要了解自己的一些 [短处/弱点/点弱/怎徒] 还要知道自己的长处。

36 We should not only understand our own [shortcomings/weaknesses/点弱/怎徒] but also know our strengths.

37 她无意中发现有一个 [哭泣/流泪/泪流/杆游] 的妇女瑟瑟发抖地蜷伏在街角。

37 She accidentally found a [crying/weeping/泪流/杆游] woman curled up trembling on the street corner.

38 正是因为李红待人 [热诚/真挚/挚真/梁点] 才让她在班里很受同学们的欢迎。

38 It is because Li Hong treats others with [sincerity/sincerity/挚真/梁点] that she is very popular with her classmates.

39 有时间你可以去感受一下 [异域/他乡/乡他/尺好] 风情和独特的他国文化。

39 If you have time, you can go to experience [foreign/a strange land/乡他/尺好] customs and unique foreign cultures.

40 全国人民坚决 [拥护/支持/持支/怪圣] 和平友好、互利共赢的思想。

40 The people of the whole country firmly [uphold/support/持支/怪圣] the idea of peace, friendship, mutual benefit and win-win results.

41 如今的小学 [教育/培养/养培/志腰] 需要将重点放在扎扎实实地打好基础上。

41 Today's primary school [education/training/养培/志腰] needs to focus on laying a solid foundation.

42 我们一直被这个令人 [困惑/费解/解费/接毒] 的问题阻扰着且毫无进展。

42 We have been blocked by this [confusing/perplexing/解费/接毒] problem and made no progress.

43 听了报告后我的心里不仅 [明朗/敞亮/亮敞/英棵] 了而且也有了新的方向。

43 After listening to the report, my heart is not only [bright/open/亮敞/英棵] but also has a new direction.

44 许多人喜欢通过品尝 [美食/佳肴/肴佳/笋祈] 的方式来让心情变得更愉悦。

44 Many people like to taste [delicacies/delicious dish/肴佳/笋祈] to make their mood more pleasant.

45 只有你认识到产生 [差别/不同/同不/友么] 的原因才有可能让二者恢复平衡。

45 Only when you recognize the cause of [differences/differences/同不/友么] can you restore balance between the two.

46 当小孩子得到了别人 [夸奖/表扬/扬表/妆查] 时会表现的很开心甚至手舞足蹈。

46 When children are [praised/commended/扬表/妆查], they will be very happy and even dance with joy.
47 我们最好能够做到不随意 [猜度/揣测/测揣/顺貂] 其他人的想法和意见。
47 We had better not casually [guess/conjecture/测揣/顺貂] the ideas and opinions of others.
48 在中秋佳节能和亲人一起 [吃饭/用餐/餐用/靠发] 是一件很幸福很温馨的事。
48 It is a very happy and warm thing to [eat/dine/餐用/靠发] with relatives on the Mid-Autumn Festival.
49 听其他人说是煤气泄露最终 [导致/引起/起引/还功] 了这场火灾的发生。
49 Others said that the gas leak finally [caused/caused/起引/还功] the fire.
50 心智尚不成熟的小孩在面对 [害怕/恐惧/惧恐/绿普] 的事物时难免会反应过激。
50 In the face of [fear/dread/惧恐/绿普] things, immature children will inevitably overreact.
51 他干了坏事以后特别害怕别人 [知道/明白/白明/本快] 这件事是他做的。
51 After doing something bad, he was particularly afraid that others would [know/understand/白明/本快] that he did it.
52 这个可爱的小男孩儿如同 [往常/平时/时平/和止] 一样向每个人打着招呼。
52 This lovely little boy greeted everyone as [usual/ordinarily/时平/和止].
53 备考的学生在梦中经常 [打颤/哆嗦/嗦哆/哽肽] 的原因是压力比较大。
53 The reason why students preparing for exams often [shiver/tremble/嗦哆/哽肽] in their dreams is that they are under great pressure.
54 如果你不能够及时 [回复/交流/流交/科卡] 信息就可能会造成很严重的后果。
54 If you cannot [reply/communicate/流交/科卡] in time, it may cause serious consequences.
55 这家开了多年的老工厂有许多 [隐患/风险/险风/统书] 需要尽快得到处理。
55 This old factory that has been in operation for many years has many [hidden dangers/risks/险风/统书] that need to be dealt with as soon as possible.
56 在这个城市里不乏有因为 [疲惫/倦怠/怠倦/奚谊] 而选择放弃的年轻人。
56 In this city, there is no shortage of young people who choose to give up because of [fatigue/burnout/怠倦/奚谊].
57 在危急关头还能保持 [冷静/镇定/定镇/觉睛] 的人大多是经历过大风大浪的。
57 Most people who can remain [calm/composed/定镇/觉睛] in critical moments have experienced great storms.
58 像他这样特别喜欢 [拍照/摄影/影摄/搞暗] 的男孩子在这个地方很少见。
58 Boys who especially like [taking pictures/photography/影摄/搞暗] are rare in this place.
59 有一些人说厨房物品 [摆放/陈列/列陈/创贩] 不得当的话会影响夫妻感情。
59 Some people say that if the kitchen items are [placed/displayed/列陈/创贩] improperly, it will affect the relationship between husband and wife.
60 生活会给你慷慨地馈赠 [甜头/好处/处好/边那] 但同时也会让你吃尽苦头。
60 Life will generously give you [sweetness/benefits/处好/边那], but it will also make you suffer a lot.
61 这个姑娘从未停止向梦想 [飞奔/疾驰/驰疾/驯廊] 的脚步和对它的热爱。
61 This girl has never stopped the steps of [dashing/galloping/驰疾/驯廊] toward her dream and her love for it.
62 良好的沟通与交流才是解除 [误会/曲解/解曲/新丹] 并且赢回信任的最好方式。
62 Good communication is the best way to resolve [misunderstandings/misinterpretations/解曲/新丹] and regain trust.

63 思念亲友并深切热爱 [故土/家乡/乡家/尺觉] 的人总难逃“近乡情怯”的现象。
63 People who miss their relatives and friends and deeply love [homeland/hometown/乡家/尺觉] can hardly escape the phenomenon of "near 乡情怯".
64 他想把这些东西全部 [隐藏/掩饰/饰掩/肤绿] 起来以防被别人发现真相。
64 He wants to [hide/conceal/饰掩/肤绿] all these things to prevent others from discovering the truth.
65 你不必因为自己拥有 [独特/奇异/异奇/兵音] 的想法而觉得自己是不正常的。
65 You don't have to feel abnormal because you have [unique/strange/异奇/兵音] ideas.
66 真理即使混杂在一堆 [假话/谎言/言谎/否领] 里也会像油浮出水面一样显现。
66 Even if the truth is mixed with a pile of [falsehoods/lies/言谎/否领], it will appear like oil floating to the surface.
67 她带着满腔 [希望/期许/许期/放消] 步伐轻盈地越过高山走到我们身边。
67 She came to us across the mountains with a heart full of [hope/expectation/许期/放消] and light steps.
68 我们应该感谢 [老师/教授/授教/谓救] 给了我们丰富的知识和看世界的眼睛。
68 We should thank [teachers/professors/授教/谓救] for giving us rich knowledge and the eyes to see the world.
69 眼前的露天 [食堂/餐厅/厅餐/层楚] 让他不禁想到过去的那段艰苦岁月。
69 The open-air [canteen/restaurant/厅餐/层楚] in front of him couldn't help but remind him of those hard times in the past.
70 他宁愿做池塘里的自由 [乌龟/王八/八王/丈义] 也不愿做受人束缚的宝马。
70 He would rather be a free [tortoise/turtle/八王/丈义] in the pond than a bound BMW.
71 这是历史上第一次使用人工 [摄像/录影/影录/数易] 记录生活中发生的点滴。
71 This is the first time in history that manual [camera/recording/影录/数易] has been used to record the bits and pieces of life.
72 青年人要有继往开来的远大 [志向/抱负/负抱/弃体] 才能成大功立大业。
72 Young people need to have great [ambitions/aspirations/负抱/弃体] to carry on the past and open up the future in order to achieve great achievements.
73 共产党的英雄 [机智/聪颖/颖聪/猿鞋] 巧妙地掩护着前来送情报的交通员。
73 The Communist Party's heroes [witty/intelligent/颖聪/猿鞋] cleverly covered the traffic officers who came to deliver intelligence.
74 聪明的人知道只有 [勤奋/努力/力努/工坚] 刻苦的拼搏才能获得成功。
74 Smart people know that only [diligence/hard work/力努/工坚] and hard work can lead to success.
75 争论情景是刺激学习和激发学习 [动机/念头/头念/东急] 的一种重要方法。
75 The scene of argument is an important method to stimulate learning and inspire learning [motivation/thought/头念/东急].
76 十年苦读为他以后的学习提供 [坚实/牢靠/靠牢/置卷] 的基础和丰富的经验。
76 Ten years of hard study provided him with a [solid/reliable/靠牢/置卷] foundation and rich experience for his future studies.
77 养成勤洗手的习惯是有效 [防止/避免/免避/乔遍] 细菌滋生的重要方法。
77 Developing the habit of washing hands frequently is an important way to effectively [prevent/avoid/免避/乔遍] the growth of bacteria.
78 我们可以通过窗户看到那两株枝叶 [浓密/茂盛/盛茂/盗茵] 的百年梧桐。

78 Through the window, we can see the two century-old plane trees with [thick/lush/盛茂/盗茵] branches and leaves.
79 这个无情无义愚蠢 [懦弱/胆小/小胆/太括] 的学生的确恨得我们咬牙切齿。
79 This heartless and stupid [cowardly/timid/小胆/太括] student really makes us gnash our teeth with hatred.
80 大街小巷里洋溢 [欢乐/开心/心开/见出] 的气氛让每个人都不由自主地笑起来。
80 The atmosphere of [joy/happiness/心开/见出] pervading the streets and alleys makes everyone laugh involuntarily.

Note. For example, in Item 1, the four preview conditions are presented as [充实/饱满/满饱/静虾]. The first two words, "充实" (fulfilling) and "饱满" (plump), are legitimate lexemes, whereas "满饱" and "静虾" are nonwords. As such, nonwords are not translated to maintain the experimental design's interference effect.
